# Palate Mucoperosteum: An Usefull Adjunct in Buccal Mucosa Reconstruction

**Published:** 2017-09

**Authors:** KN Manjunath, Veena P. Waiker

**Affiliations:** Department of Plastic and Reconstructive Surgery, MSRMC, Bengaluru, Karnataka, India

**Keywords:** Mucoperosteum, Palate, Versatile reconstructive

## Abstract

**Background:**

Palate is a complex structure separating oro- and nasopharynx. However, reconstruction of the defects of palate is much simpler because of the versatile mucoperiosteal flaps. Here, we present our experience of palatal mucoperiosteal flap used in different situations.

**Methods:**

Fifteen patients of palatal as well as buccal mucosa defects were reconstructed using either free or pedicled mucoperiosteum.

**Results:**

All patients recovered well. No flap loss or secondary procedure were required.

**Conclusion:**

Success in Reconstruction of the palatal defects depends on creation of good nasal as well as buccal mucosal lining. The rich vascular macronet in the palatal mucosa makes it an ideal donor site for local reconstruction. The mucoperiosteum harvested either as a free graft or as pedicled flap serves the purpose well leaving no donor site deformity.

## INTRODUCTION

Palate is a complex structure both anatomically and functionally, comprising of bone, muscle and mucosa. Functionally both hard and soft palate, together serve as one unit to separate nasopharynx and oropharynx. Such separation is important for articulation of speech and deglutition.^1^ Although the structure and function of palate (both hard and soft) is complex, reconstruction of defects of palate is much simpler because of the versatile mucoperiosteal flaps.^2^ The mucoperiosteum over the bony palate is highly vascular and has enormous potential to heal. The bare bony frame work epithelises leaving no donor site deformity. All these features make it the best available tissue for reconstruction of local as well as adjacent buccal mucosa.^3^

## MATERIALS AND METHODS

Ten patients of palatal as well as buccal mucosa defects were reconstructed using either free or pedicled mucoperiosteum. (i) Two patients were case of isolated Group-2 cleft palate, in which Wardill-Kilner-Veau pushback palatoplasty was done. The nasal layer breached during dissection and suturing. Hence, repair of nasal lining at the junction of hard and soft palate could not be achieved. The two mucoperiosteal flaps were intact. The nasal mucosa was reconstructed by flipping the left mucoperiosteal flap with mucosa facing nasal cavity and it was sutured to the bony palate.

**Fig. 1:**
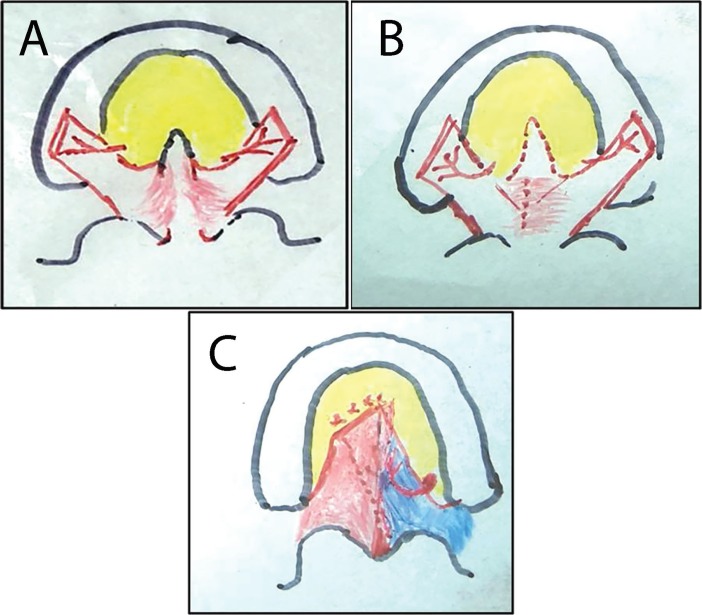
**A.** Cleft palate (both sides of mucoperiosteum were elevated). **B.** Rent in the nasal lining. **C. **Left mucoperiosteal flap used for nasal lining and right for buccal lining

**Fig. 2:**
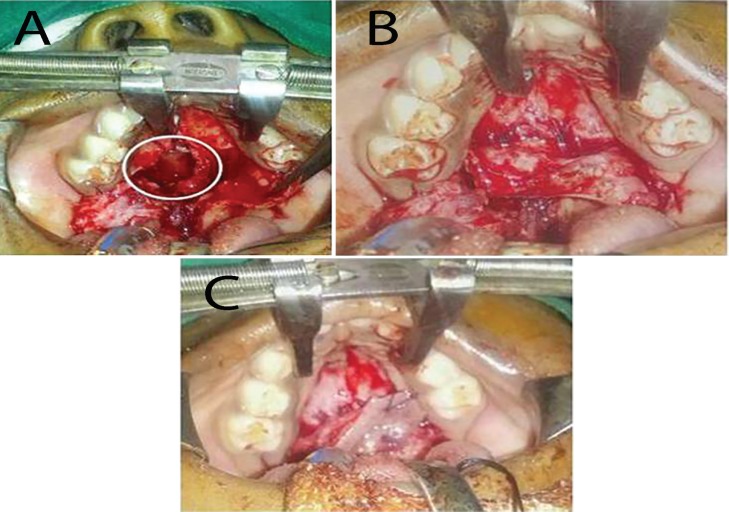
**A.** Dehiscence of nasal mucosal lining. **B.** Left side mucoperiosteal flap in-turned. **C.** Right mucoperiosteal flap draped over

On top of this layer the right mucoperiosteal flap was transposed and sutured to create the oral mucosal layer (Figure 1 and 2). Thus we could achieve reconstruction of both nasal and oral mucosal layers with the two mucoperiosteal flaps at this critical junction. (ii) Four patients had minor salivary gland tumour at the junction of hard and soft palate. The lesion extended on to the pedicle of mucoperiosteal flap on one side. In these cases, one half of the left mucoperiosteal flap based on the greater palatine artery was used to resurface the exposed intact nasal mucosa on right side.

Four patients had buccal mucosal defect following excision of early malignancy. In these patients, mucosa was replaced with free grafts (Mucoperiosteal) from the intact palate. The free mucoperiosteal graft took very well and functioned as mucosa after the wound healed.

Pedicled flaps as well as free grafts healed very well without any morbidity. Palatal donor site healed by epithelisation and had no further deformity.

## RESULTS

Pedicled flaps as well as free grafts healed very well without any morbidity. Palatal donor site healed by epithelisation and had no further deformity. Initially when pedicled flap was used, there was speech disturbance which improved later.

## DISCUSSION

Palate is a complex structure. On either side of palate, there are cavities which have major functions (Nasal cavity- speech, oral cavity– speech and mastication). The separating structure has thin bone, covered on either side by mucosa anteriorly. As we move posteriorly the rigid hard palate becomes dynamic soft palate^4^ towards the in posterior region. This dynamicity is due to the musculature of the soft palate.^5^ Trauma and scarring of the soft palate mucosa may lead to tethering and hamper speech and deglutition, hence its mucosa can neither be harvested nor be used for any reconstruction.^6^^,^^7^

The buccal mucosa, as a donor site for grafts in itself is very versatile, as it heals on its own and leaves no donor site deformity. However, the mucoperiosteum of hard palate, is supplied by greater palatine artery is highly vascular. In view of its versatility it can either be harvested as a free graft or pedicled flap.^6^^,^^8^ Pedicled mucoperiosteal flaps can either be islanded or Peninsular flap.^9^^,^^10^ The Greater palatine artery entering the mucoperiosteum at the palatine foramen forms the major supply and one pedicle can sustain most of the mucoperiosteum over the palate. In two of our cases of complete cleft of secondary palate, during the Wardill-Kilner-Veau (V-W-K) repair, the nasal mucosa breached and resulted in fistula at the junction of hard and soft palate. The thinness of nasal mucosa and less tonicity makes it very difficult to suture and as literature shows fistula is inevitable in inexperienced or even in case of experienced surgeons.^8^ The nasal and oral mucosa was deficient. Hence the adjacent mucoperiosteal flaps were used to create both nasal and oral mucosa. Creating a good nasal lining is an important step in cleft palate repair and double layered closure is the mainstay for a successful cleft palate repair.^8^ Every effort should be given to create tough nasal mucosal layer. The thinness of nasal mucosa and less tonicity makes it very difficult to suture and fistula is inevitable in inexperienced/ even in case of experienced surgeons.^8^

On table nasal mucosal fistula as a small perforation can be left alone to contract and heal but if large, then a salvage procedure to recreate nasal lining is a mandate. Variety of flaps has been used as salvage procedure. Ipsilateral vomerine flap is one. Buccal mucosal flap is another choice.^11^ But in our case, we used one side palatine mucoperiosteal flap for nasal lining and other for oral lining. Both the flaps used to create palatal separation healed without complication and had good speech outcome.

The vascular capillary network (Vascular macronets) allows whole of the mucoperiosteum to be elevated based on one greater palatine artery and also as islanded pedicle flap.^12^ In two of our cases of salivary gland tumor, post-excision defect was situated at the junction of hard and soft palate. In one case, the challenge was to recreate the mucosal lining without altering soft palate function. Here we used the whole of mucoperiosteal islanded flap based on one greater palatine artery to cover the area.

Since 1977, island flap for palatal reconstruction is used with 95% success rate^14^.But the posterior reach of islanded palatine flap is difficult, due to the bony canal through which the pedicle emerges. But breaking the posterior wall of the palatine foramina makes the reach possible.^13^ The donor site heals completely in 2-3 weeks without contractures or compromising function of the palate.^14^^,^^15^

Free graft in our case was used for a small carcinoma in situ, where the maximum mucosal defect was 3×4 cm. This has the advantage of being local tissue and hence it does not require any other donor area for harvesting. The donor site also granulates and heals well by epithelisation.In our adult patient there was no donor site morbidity. Free graft has been used in recalcitrant pharyngeal wall stenosis.^16^ The Results of palatal free grafts were superior to skin grafts. Whereas buccal mucosal grafts as skin grafts resulted in secondary contracture and the area had less pliability. Small defects in oral cavity can be left alone for secondary healing or skin grafted. However these have disadvantages like unpredictable contracture, initial malodorous discharge and pooling of saliva.^17^ Similarly use of mucoperiosteal flap did not result in donor site deformity and was native to the tissue. But the limitation was the size of graft available. Larger defects definitely need distant/regional flap.^18^^-^^21^ In all our cases, there was no flap loss/necrosis. Even in free grafts, we had excellent outcome. There was absolutely no donor site deformity.

Palate is a complex structure both functionally and anatomically. Successful reconstruction of palatal defects depends on creation of good nasal as well as buccal mucosal lining. The rich vascular macronet in the palatal mucosa makes it an ideal donor site for local reconstruction. The mucoperiosteum harvested either as a free graft or as pedicled flap serves the purpose well leaving no donor site deformity.

## CONFLICT OF INTEREST

The authors declare no conflict of interest.
